# Comparison of one versus two maxillary molars distalization with iPanda: a finite element analysis

**DOI:** 10.1186/s40510-021-00356-6

**Published:** 2021-05-03

**Authors:** Kamontip Sujaritwanid, Boonsiva Suzuki, Eduardo Yugo Suzuki

**Affiliations:** Department of Orthodontics, Faculty of Dentistry, Bangkokthonburi University, 16/10 Leabklongtaweewatana Rd., Taweewatana District, Bangkok, 10170 Thailand

**Keywords:** Class II malocclusion, Finite element method, Maxillary molar distalization, Miniscrew

## Abstract

**Background:**

The purpose of this study was to compare the stress distribution and displacement patterns of the one versus two maxillary molars distalization with iPanda and to evaluate the biomechanical effect of distalization on the iPanda using the finite element method.

**Methods:**

The finite element models of a maxillary arch with complete dentition, periodontal ligament, palatal and alveolar bone, and an iPanda connected to a pair of midpalatal miniscrews were created. Two models were created to simulate maxillary molar distalization. In the first model, the iPanda was connected to the second molar to simulate a single molar distalization. In the second model, the iPanda was connected to the first molar to simulate “en-masse” first and second molar distalization. A varying force from 50 to 200 g was applied. The stress distribution and displacement patterns were analyzed.

**Results:**

For one molar, the stress was concentrated at the furcation and along the distal surface in all roots with a large amount of distalization and distobuccal crown tipping. For two molars, the stress in the first molar was 10 times higher than in the second molar with a great tendency for buccal tipping and a minimal amount of distalization. Moreover, the stress concentration on the distal miniscrew was six times higher than in the mesial miniscrew with an extrusive and intrusive vector, respectively.

**Conclusions:**

Individual molar distalization provides the most effective stress distribution and displacement patterns with reduced force levels. In contrast, the en-masse distalization of two molars results in increased force levels with undesirable effects in the transverse and vertical direction.

## Background

Maxillary molar distalization has become an important approach for the treatment of class II malocclusions. It allows the movement of first molars to occlude into a class I relationship, thus facilitating the correction of crowding and reducing the overjet [1, 2].

Several tooth-borne distalizing devices have been introduced to correct class II malocclusion [3]. However, undesirable dental anchorage loss, represented by the mesial tipping and extrusion of the maxillary premolars, with an increase of anterior crowding, limited their application [4]. With the development of orthodontic miniscrew implants to provide absolute anchorage, it became possible to perform efficient maxillary distalization without compromising dental anchorage [5].

Successful distalization of the maxillary first molar alone, before the eruption of the second molar in young patients, has been reported by several authors [6]. However, in adult patients, the distalization of the maxillary first molar alone is only possible followed by the distalization of the second molars or extraction of the second molars [7].

The use of *“*en-masse” distalization of both first and second maxillary molars aided by miniscrew-supported appliances has been proposed to reduce the total treatment duration [8]. However, to achieve sufficient amounts of distalization, an increase in the level of forces (200–500 g) is required [9, 10]. Consequently, the precise three-dimensional control of tooth movements, such as tipping, extrusion, and rotations, has become critical [11]. Moreover, if the heavy distalizing forces are applied throughout the first maxillary molar, to perform the en-masse distalization, an unpredictable movement of the second molar can be expected [12].

Recently, an innovative miniscrew-supported distalization device in the palatal area so-called the “indirect Palatal Anchorage and Distalization Appliance” (iPanda) was introduced to allow controlled movement of maxillary molar teeth in three-dimension during distalization. Since the maxillary molars were indirectly anchored to the midpalatal miniscrews, maxillary molar distalization was possible without compromising dental anchorage [13]. Although the clinical study with iPanda demonstrated a well-controlled bodily distalization of the first maxillary molars alone [7, 13], the effects of the en-masse distalization of both first and second maxillary molars have not been clarified. Moreover, the biomechanical effect of distalization forces on the iPanda is unknown.

Several clinical and in vitro studies have been conducted to investigate the performance of different distalization appliances and to evaluate the type, amount, and rate of tooth movement [1, 2]. However, little information regarding the pattern of the mechanical response on load application generated in the tissue surrounding teeth can be generated during clinical studies. Therefore, to overcome such limitations, the use of finite element analysis (FEA) has become a common and reliable approach to quantitatively assess the stress and strain distribution generated in response to various biomechanical settings in orthodontics [14, 15].

Therefore, the purpose of this study was to compare the stress distribution and displacement patterns of the one versus two maxillary molars distalization with iPanda and to evaluate the biomechanical effect of distalization on the iPanda using the finite element (FE) method.

## Materials and methods

### Model creation

A FE model was created from computed tomography images of a maxillary arch which includes maxilla and complete permanent dentition except for the third molar of an adolescent (slice thickness, 1.0 mm). A three-dimensional computer-aided design program (NX version 10.0, Siemens PLM Software, Plano, TX, USA) was used to construct the model and standardize according to Wheeler [16] and Andrews [17].

The model consisted of virtually constructed teeth, 0.2 mm of periodontal ligament [18], palatal bone, alveolar bone. The thickness of the cortical bone was determined according to Farnsworth et al. [19]. A 0.018-inch Roth-prescription brackets, 0.016 × 0.022” stainless steel archwire, and an iPanda, which was composed of a 0.9-mm-diameter palatal bar cobalt-chrome, iPanda bracket, and a pair of miniscrews (1.6 × 6 mm) in the midsagittal region were constructed, following Suzuki and Suzuki protocol [13]. All materials were considered homogenous, isotropic, and linear elasticity.

The model and appliances were imported into LS-DYNA (version 971, Livermore Software Technology Corporation, Livermore, CA, USA) to produce a tetrahedral FE mesh; the maxilla including the teeth and alveolar bone meshed into 0.5-mm tetrahedrons.

**Fig. 1:**
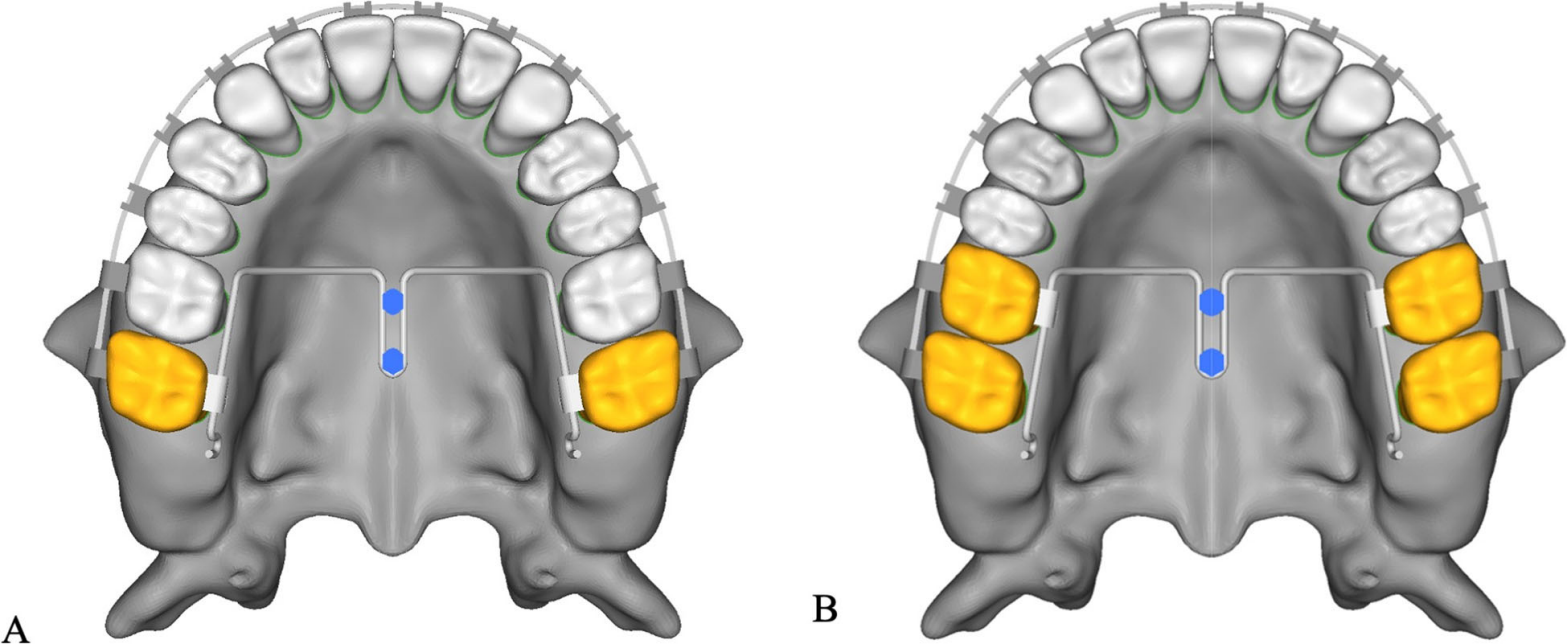
Experimental conditions: **a** The iPanda was attached to the second molar to simulate one molar distalization. **b** The iPanda was attached to the first molar to simulate two molars distalization

Two set-ups models of molars distalization with iPanda were constructed to simulate maxillary molar distalization: In the first model, the iPanda was connected to the second molar to simulate a single molar distalization (Fig. 1a). In the second model, the iPanda was connected to the first molar to simulate the en-masse of the first and the second molar distalization (Fig. 1b). Materials’ properties (Young’s modulus and Poisson’s ratio) were determined according to previous studies (Table 1) [20].

**Table 1:** Young’s modulus and Poisson’s ratio for various materials

Material	Young’s modulus (MPa)	Poisson’s ratio
Cortical bone	13700	0.26
Cancellous bone	1370	0.30
Tooth (dentin)	19613.30	0.15
Periodontal ligament	0.6668	0.49
Miniscrew	105000	0.33
Stainless steel wire	200000	0.30

The von Mises stress is used to predict the failure of material according to the von Mises yield principle, which states that yielding of the material occurs when the von Mises stress exceeds the yield strength in tension [21].

### Three-dimensional coordinate system and boundary conditions

A three-dimension coordinate system was constructed with the *x*-axis corresponding to the bucco-palatal direction, the *y*-axis the anteroposterior direction, and the *z*-axis the superior-inferior direction. A +*x* value was defined as the buccal direction, +*y* as the distal direction, and +*z* as the apical direction. The displacements of the teeth were calculated by applying the *x*, *y*, and *z* coordinates at the mesiobuccal, distolingual cusp, and mesiobuccal root apex of the molars. The model was constrained at the nasal floor side and posterior border of the maxillary bone in all directions. Surface-to-surface interactions between adjacent teeth were used to create the contact interfaces. The contacts between the brackets and the wires were presumed to be frictionless.

### Force simulation

**Fig. 2:**
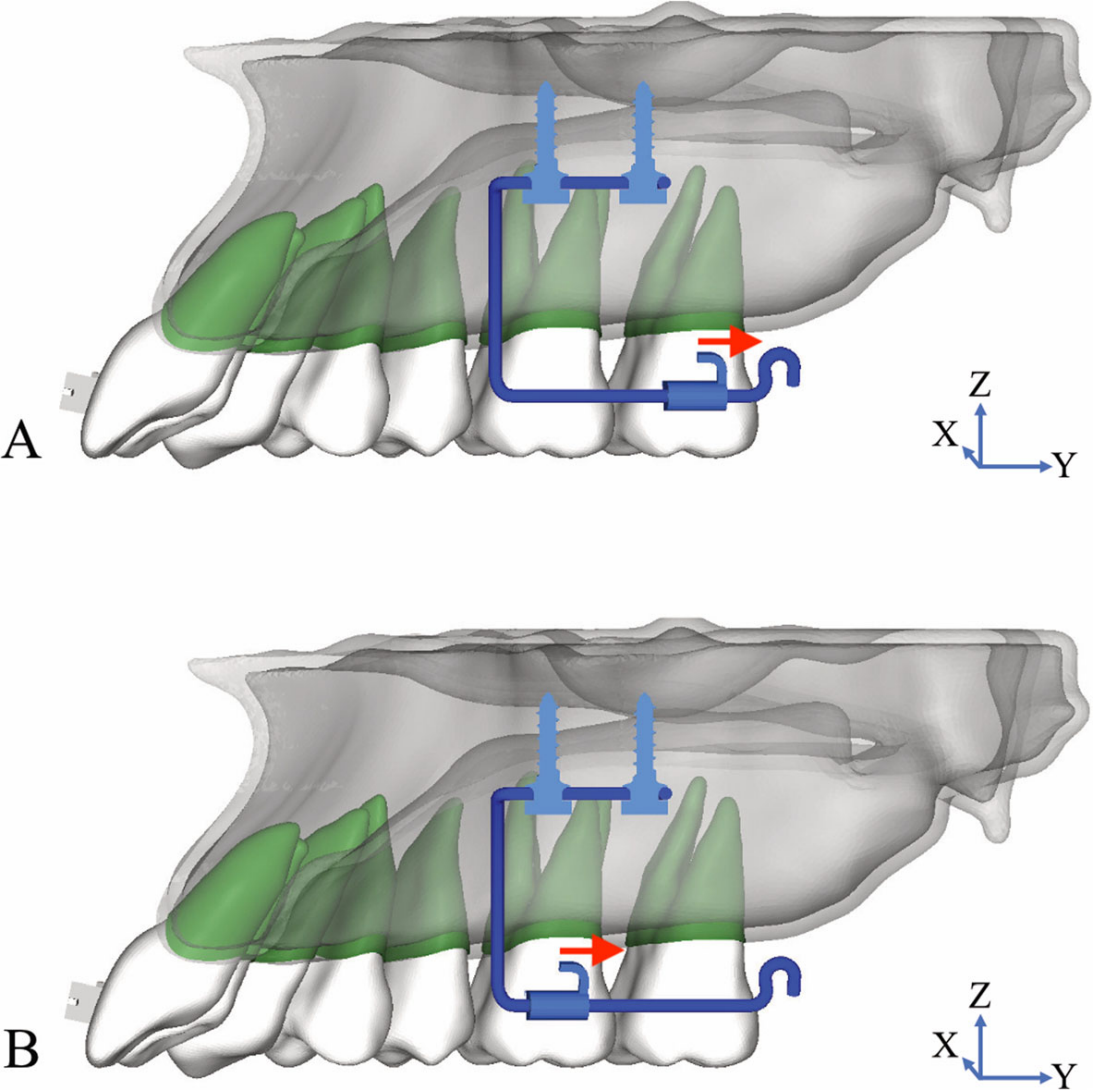
Applied distalization force parallel to *y*-axis from the tube of the iPanda on the palatal side to the hook (**a**) one molar distalization (**b**) two molars distalization

Distalization was simulated using various forces (50 g, 100 g, and 200 g). Force direction was applied from the hook of the palatal bracket to the hook at the distal end of the iPanda which is parallel to the occlusal plane (Fig. 2a, b).

Maximum von Mises stress, stress distribution pattern, and displacement of the posterior maxillary dentition on each model were analyzed. The stress was calculated and presented in value (MPa) and colorful contour bands, where different colors represent different stress levels in the deformed state.

In the present study, the simulation of distalization was performed on a single model that was created directly from the pixels of a CT image of one subject; therefore, only descriptive analysis was used.

## Results

### Stress distribution pattern

**Fig. 3:**
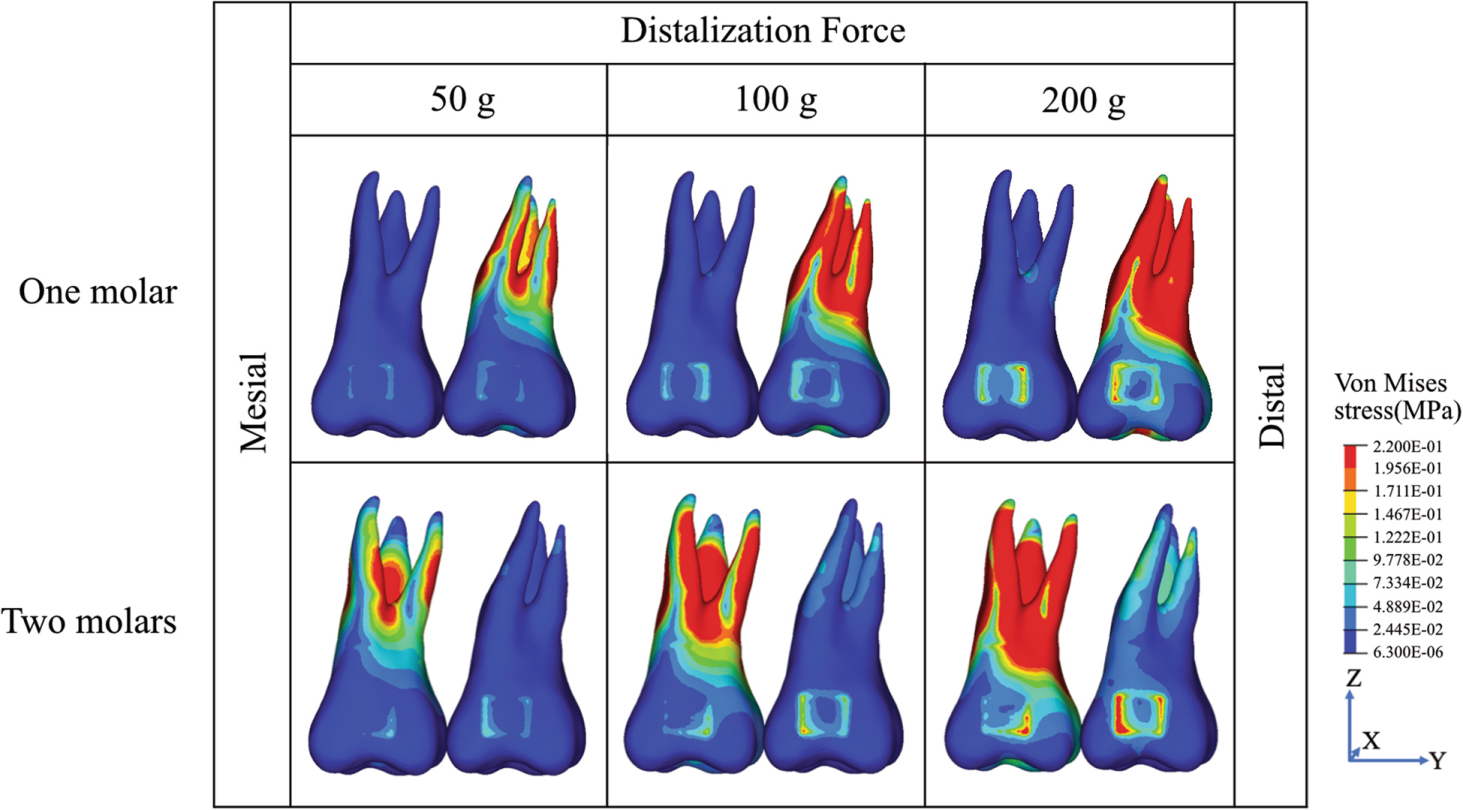
The von Mises stress distribution of one molar distalization and two molars distalization

For both models, one molar and two molars distalization, the pattern of stress distribution was similar. The stress was concentrated mainly in the furcation area and along the distal surface of all roots (Fig. 3)

However, for the two molars distalization, the stress observed in the second molar was minimal and mainly concentrated along the mesial surface of the roots (Fig. 3). Moreover, the FEA showed that the stress in the first molar was approximately 10 times higher than in the second molar (Table 2).

**Table 2:** Value of maximum von Mises stress in the PDL of posterior teeth (MPa)

Force (g)	Von Mises stress (MPa)									
	First molar					Second molar				
	*M*	*D*	*B*	*P*	Furcation	*M*	*D*	*B*	*P*	Furcation
One molar										
50	0.0002	0.0002	0.0002	0.0002	0.0001	0.0048	0.0033	0.0030	0.0046	0.7600
100	0.0004	0.0005	0.0005	0.0005	0.0002	0.0096	0.0067	0.0059	0.0091	1.5200
200	0.0006	0.0010	0.0010	0.0010	0.0003	0.0192	0.0136	0.0116	0.0180	3.0400
Two molars										
50	0.0047	0.0041	0.0031	0.0047	0.0041	0.0003	0.0005	0.0004	0.0005	0.0001
100	0.0095	0.0082	0.0063	0.0095	0.0082	0.0006	0.0009	0.0009	0.0009	0.0002
200	0.0189	0.0164	0.0125	0.0189	0.0163	0.0011	0.0018	0.0018	0.0018	0.0004

**Fig. 4:**
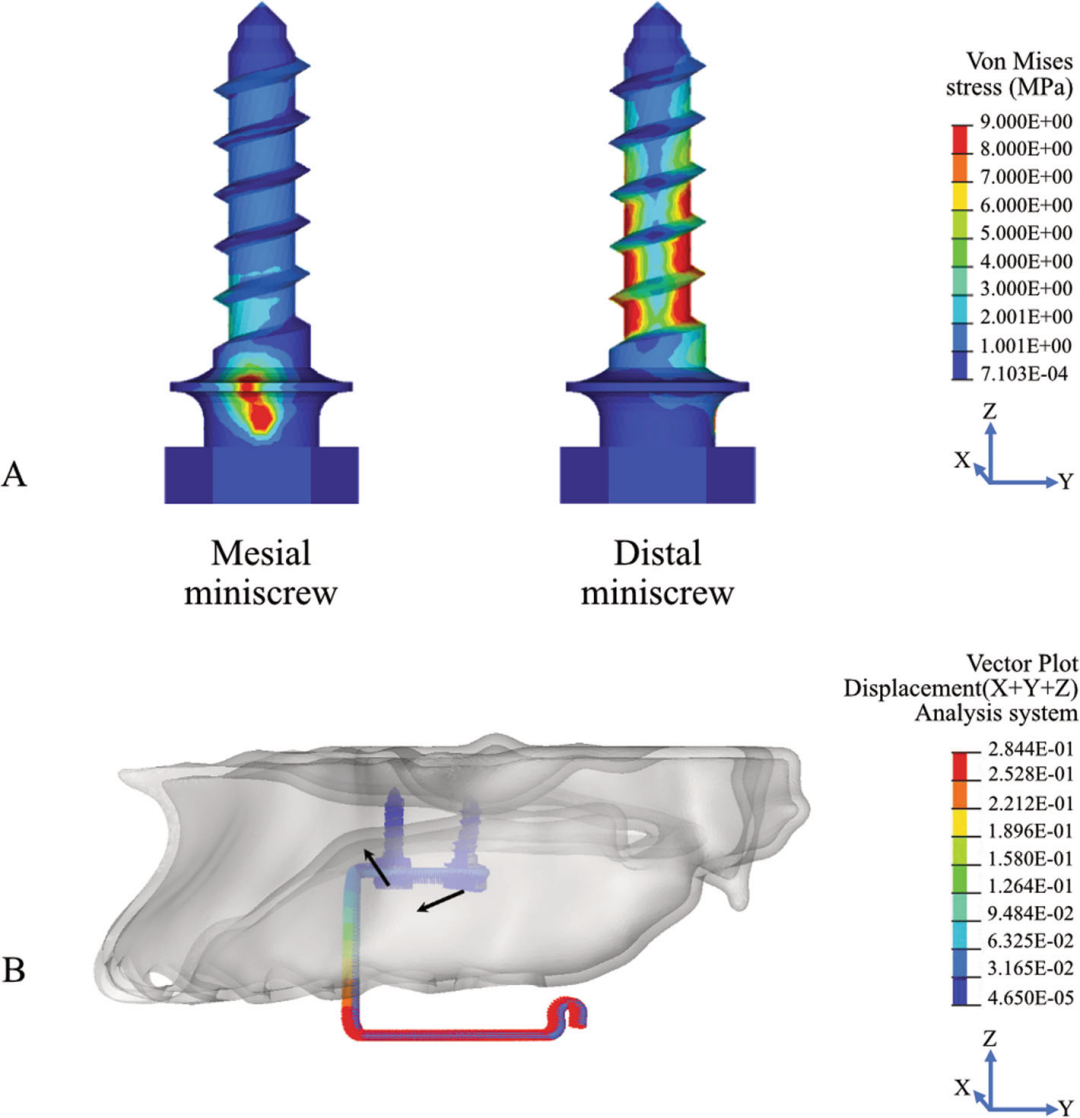
**a** The von Misses stress in mesial and distal miniscrew. **b** The direction of miniscrews displacement. The mesial miniscrew had mesial tipping with intrusive vectors, and the distal miniscrew had mesial tipping with extrusive vectors

For both distalization models, the stress distribution showed a particular pattern of stress concentration in the pair of miniscrews (Fig. 4, Table 3).

**Table 3:** Maximum von Mises stress in the miniscrews (MPa)

Force (g)	Von Mises stress (MPa)			
	One molar		Two molars	
	Mesial miniscrew	Distal miniscrew	Mesial miniscrew	Distal miniscrew
**50**	3.5232	17.6845	3.8221	18.0311
**100**	5.7414	37.4301	9.8532	38.0553
**200**	9.3562	79.2226	25.4011	80.3171

A different pattern of stress distribution between the mesial and distal miniscrew was observed. High-stress concentration was observed on the mesial and distal aspects from the cervical third to the middle third of the distal miniscrew. In contrast, the high-stress concentration was observed in the platform of the mesial miniscrew. Moreover, the stress concentration on the distal miniscrew was six times higher than the mesial miniscrew.

### Displacement pattern

For both models, one molar and two molars distalization, a similar initial displacement pattern in the transverse dimension (*x*-axis) was observed. Buccal tipping of the molar was observed in both models. The amount of buccal tipping displacement increased with the load increment. 50 g loading resulted in the least amount of buccal tipping for both models.

In the anteroposterior dimension (*y*-axis), a characteristic initial displacement pattern and amounts between one and two molars distalization were observed. The one molar distalization model promoted the greatest amount of distalization compared to the two molars distalization in all loading conditions.

However, the one molar distalization produced a higher amount of initial molar tipping than the two molars distalization model. In contrast, the two molar distalization produced a higher distobuccal crown rotation compared to one molar distalization model. 50 g loading resulted in the least amount of distobuccal rotation. Moreover, for two molars distalization, only a slight amount of distal movement of the second molar was observed in all loading conditions.

**Fig. 5:**
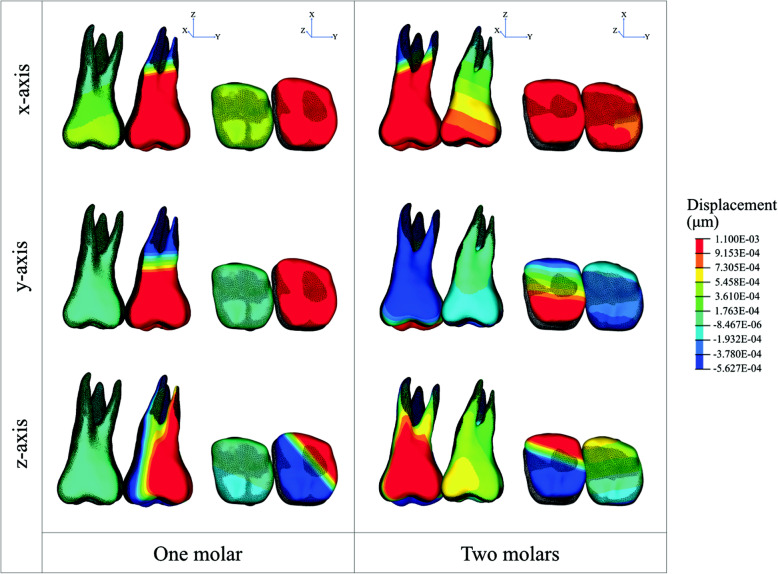
Contour image of the displacement of **a** one molar distalization, **b** two molars distalization in *x-*, *y-*, and *z*-axes. The black mesh showed the original position. The color image shows the movement of the teeth after applied 200 g distalization (× 200 times magnification)

In the vertical dimension (*z*-axis), the initial displacement pattern showed characteristic differences between one and two molars distalization. The one molar distalization model produced a similar pattern of displacement between mesiobuccal and distolingual cusps. In contrast, two molars distalization generated a higher distolingual cusp displacement (extrusion) compared to the mesiobuccal cusp, causing the outward tilting of the first molar (Fig. 5, Table 4).

**Table 4:** The initial displacement of the first and second molar (μm)

Force	Tooth	Landmark	Displacement (μm)					
			***x***-axis		***y***-axis		z-axis	
			One molar	Two molars	One molar	Two molars	One molar	Two molars
50 g	First molar	Mesiobuccal cusp	0.10	1.92	0.03	0.39	0.19	− 0.12
		Palatal root	0.01	− 0.70	0.01	− 0.20	0.01	− 0.05
		Distolingual cusp	− 0.01	2.18	0.04	0.81	− 0.01	− 0.84
	Second molar	Mesiobuccal cusp	1.73	0.36	1.64	0.16	− 0.47	0.09
		Mesiobuccal root	− 0.63	− 0.04	− 0.35	0.07	− 0.09	0.07
		Distolingual cusp	1.72	0.30	1.78	− 0.06	− 0.55	− 0.05
100 g	First molar	Mesiobuccal cusp	0.20	3.82	0.06	0.81	0.23	− 0.24
		Mesiobuccal root	0.02	− 1.39	0.02	− 0.40	0.02	− 0.10
		Distolingual cusp	− 0.02	4.43	0.08	1.64	− 0.02	− 1.67
	Second molar	Mesiobuccal cusp	3.62	0.72	2.59	0.32	− 0.56	0.17
		Mesiobuccal root	− 1.34	− 0.08	− 0.47	0.13	− 0.33	0.15
		Distolingual cusp	4.17	0.59	2.82	− 0.11	− 0.70	− 0.11
200 g	First molar	Mesiobuccal cusp	0.40	7.60	0.12	1.68	0.28	− 0.48
		Mesiobuccal root	0.04	− 2.76	0.04	− 0.80	0.04	− 0.20
		Distolingual cusp	− 0.04	9.00	0.16	3.32	− 0.04	− 3.32
	Second molar	Mesiobuccal cusp	7.57	1.44	4.09	0.64	− 0.67	0.32
		Mesiobuccal root	− 2.85	− 0.16	− 0.63	0.24	− 1.21	0.32
		Distolingual cusp	10.11	1.16	4.47	− 0.20	− 0.89	− 0.24

*X* transverse axis, *Y* anteroposterior axis, *Z* vertical axis. A *+x* value was defined as the buccal direction, *+y* as the distal direction, and *+z* as the apical direction

A particular pattern of displacement between the mesial and distal miniscrews was observed. While the mesial miniscrew had mesial tipping combined with intrusive vectors, the distal miniscrew had mesial tipping combined with extrusive vectors (Fig. 4).

## Discussion

The possibility of “total arch distalization’ or en-masse distalization of the molars for the correction of class II malocclusions remains a challenge in orthodontics. The main advantage of this approach is the possibility of reducing the overall treatment duration while simplifying the distalization steps [8]. However, the distalization of several teeth requires an increase in the force magnitude that might cause undesirable biomechanical effects. Therefore, the purpose of the present study was to compare the biomechanical effects of one versus en-masse two molars distalization using the iPanda.

### Stress distribution pattern

For the one and two molars molar distalization models, the stress was concentrated at the furcation area and along the distal surface of all roots. This stress distribution pattern showed that the distalizing force provided by the iPanda can be advantageous for a controlled movement of the molars. This result is in agreement with a previous clinical study that showed the bodily distalization of a single molar with the iPanda [7, 13]. Moreover, since the center of resistance of the molar is located within the furcation area, the desirable resultant distalization vectors should pass to the level of the molar’s center of resistance to minimize the distal tipping of the molars [22]. The stress distribution at the furcation area and along the distal surface of the roots suggests the controlled distal movement of the molar.

In the present study, the use of 50 g force to distalize the single molar was shown to be beneficial to promote homogeneous stress distribution along with the distal surface of the molar roots during distalization with minimal side effects. Moreover, a single tooth movement generates a more simplified force system with reduced undesirable effects in adjacent teeth. The result of this study is in agreement with Kinzinger et al. [6] who investigated the effect of tooth eruption in a composed system during distalization. In their study, it was concluded that the stage of development of the third molar influenced the pattern of tooth movements, such as tipping and extrusion.

For en-masse two molars distalization, the increase in the load at the first molar was not sufficient to generate an increase in the stress along with the distal surface of roots of the second molar that would promote their distalization. FEA showed that the stress in the first molar was approximately 10 times higher than in the second molar, thus indicating that the force delivered to the second molar would not be sufficient to cause the distalization of the second molar. The difference in stress distribution between the first and second molars indicated that the force transfer from the first to the second molar is far beyond ideal; therefore, the force system might be adjusted to allow optimized distalization. The results are in agreement with the previous article. The study Ammoury et al. [23] compared the effect of direct versus indirect anchorage for en masse distalization. They concluded that 150 g is not enough for efficient distalization, since it resulted in low stresses and displacements at the molars. Therefore, the need for a heavier force for the initial molar movement is necessary.

Based on the results of the present study, the suggested approach for the distalization of two molars would be the simultaneous distalization of the first molar with the iPanda (100 g) combined with the distalization of the second molar aided by an open coil spring (50 g) applied with the orthodontic appliance. With this design, both molars are simultaneously distalized with reduced force levels. The distalization force applied to the second molar provided by the open coil spring (50 g) generates undesirable mesialization forces to the first molar. However, this mesialization force is neutralized with the iPanda providing direct distalization forces (100 g) to the first molar, thus resulting in the distalization of both first and second molars. This suggested design might improve the results in terms of biomechanical performance. However, further studies need to be performed to evaluate its effectiveness.

In the present study, a characteristic stress distribution pattern between the mesial and distal miniscrew was observed. A higher-stress concentration was found in the distal miniscrew compared to the mesial miniscrew. The results are in agreement with the previous iPanda FEA study [24]. Moreover, although the stress distribution pattern of the miniscrews in both models was relatively similar, the amount of stress in the one molar was lower than the two molar distalization.

The stress distribution pattern showed the role of each miniscrews that resisted the distalization force in three dimensions. The stress in the mesial and distal surface of the threaded part of the distal miniscrew showed the resistance of the miniscrew to the distalization force in the sagittal direction during iPanda activation. While the stress in the mesial miniscrew which shows at the lateral side of the miniscrew platform showed resistance to the vertical intrusive force. The stress in the distal miniscrew was approximately six times higher than in the mesial miniscrew, thus indicating that the distal miniscrew is more prone to higher stress and more prone to failure.

Although the stress distribution in the miniscrews in both models followed the same pattern, the relationship of the position of the mesial miniscrew and the moving tooth might have effects on the stress in the tooth.

### Displacement pattern

Regarding the displacement pattern for one molar distalization, the initial displacement analysis demonstrated a large amount of molar distalization with a small amount of undesirable distal and buccal crown tipping. This result is in agreement with previous clinical studies of molar distalization [13]. The displacement pattern exhibited by one molar distalization under 50 g load represents the desired tooth movement during the distalization of a molar with reduced side effects. Moreover, the results also support the efficiency of the iPanda for the three-dimensional control of the tooth movement during distalization.

In contrast, for the en-masse two molars distalization, a minimum amount of distalization combined with a high tendency towards distobuccal rotation, buccal tipping, and outward tilting of the first molar was observed. Although the iPanda is effective for a single molar distalization, the presence of a second molar generates undesirable resistance to the movement of the first molar which resulted in undesirable moments. Moreover, only a minimal amount of distal movement of the second molar was observed despite a load increase. The main explanation is that the load up to 200 g applied to the first molar was not enough to be transferred to the second molar. According to the results, the load applied to the first molar should higher than the load used in the present study to achieve a sufficient amount of distalization of the second molar. Consequently, the high levels of force ultimately generate various undesirable biomechanical effects, such as uncontrolled tipping and extrusions [25].

In the present study, both one and two molars distalization models exhibited consistent buccal movement of the molars. The first explanation for this buccal movement is the design of iPanda’s power arms that are straight and do not conform with the curved ovoid shape of the skeletal base. Adjustments in the direction of the power arms to conform to the dental arch form are important to minimize this problem. The second explanation is the height of force application, since the distalizing force is applied coronally to the center of resistance of the molars, therefore facilitating the buccal crown tipping of the molars. This results are in agreement with Yu et al. who described the displacement pattern of distalization first and the second molar simultaneously by a palatal plate in FEA, which shows that the first molar moved more widen laterally than distalized only first molar. A more heavy archwire would reduce distortions and minimize these effects [26].

In the present study, for both one and two molars distalization models, a minimal amount of extrusion of the molars was observed. This also can be explained by the height of force application coronally to the center of resistance of the molars and to the vector of force application parallel to the occlusal plane. Therefore, resulting in extrusion displacement. To avoid this effect, adjustments in both height and vector of force application should be performed. Our findings are in accordance to study of Yu et al. which use a palatal plate with various heights of the distalization vectors [26]. The authors further reported that the distalization force close to midpalatal suture, the effect of extrusion was replaced with intrusion and bodily movement might be achieved [26].

The en-masse distalization of first and second maxillary molars has been attempted by several authors with contrasting results [27, 28]. It has been reported that the presence of the second molars increases the duration of the distalization and produces more tipping of the second molars with anchorage loss [28]. In contrast, Bussik and Mcnamara reported that the presence and position of the second maxillary molar did not influence the amount and the type of the maxillary first molar distalization [29].

Moreover, the patient’s age and the stage of the second molar development play an important role in the amount and pattern of molar distalization [6]. Most of the studies that were performed in young patients, when the second molars were not completely erupted, the en-masse distalization of the first and second molars had been successfully performed [4, 30]. Comparing distalization effects according to molar eruption stage had observed that the distalization of the first molar with an unerupted second molar was 20% greater than that when the second molar was erupted [30]. However, it is also suggested that the most efficient timing when using an MPAP is after the full eruption of the second molar, since it decrease the mesial-in rotation of the molars. Therefore, confirming the strong influence of the presence of an erupted tooth on the path of distalization. A different approach for en-masse distalization using a sequential distalization pattern with an individual distalization of the second molar should be employed to obtain the maximum benefits of the en-masse distalization.

In the present study, a particular pattern of displacement between the mesial and distal miniscrews was observed. A couple of force was created with the pair of miniscrews, with the mesial miniscrew receiving an intrusion force and the distal miniscrew receiving an opposing extrusion force [31]. This system allows for the creation of a stable skeletal anchorage to withstand the high magnitude force that is required to perform en-masse distalization forces. Moreover, the pair of miniscrew implants of the iPanda is often placed in the midpalatal suture area, which is often regarded as the preferred site for miniscrew placement in the maxilla for its keratinized soft tissue and sufficient cortical bone [32]. Consequently, high stability values are often obtained with miniscrews placed in the midpalatal suture [33].

Our results suggest that the distalization of one molar is the most efficient treatment approach to obtain a controlled distalization of a molar, with the advantage of applying relatively low forces with reduced dental undesirable effects. On the other hand, the en-masse distalization of two molars requires high distalization force values that produce increased distal tipping, buccal tipping, and extrusion of the first molar. Additionally, the en-masse distalization produces minimal effects for the second molar.

However, the extension of the interpretation for a clinical situation should be considered with limitations. Since the results of FEM just explained the initial effect of stresses and tooth displacements within the PDL space before bone remodeling in one condition, the FEM presents intrinsic limitations. Therefore, evaluation of biomechanical effects in the case of anatomical variation, such as different bone stiffness or bone thickness, might result in different outcomes [23]. Moreover, improvement of the distalization force system should be performed to allow adequate force distribution during the en-masse distalization of the molars. Therefore, further studies are necessary to elucidate the optimum force system to perform the en-masse molar distalization.

## Conclusions

1. Distalization of one molar with the iPanda generates the most effective stress distribution and displacement patterns, therefore, ideal for controlled movement. Moreover, reduced force levels are sufficient for individual molar distalization.
2. In contrast, the en-masse distalization of two molars with the iPanda, requires a high increase in the force levels to be applied to the first molar to allow the transfer of distalizing forces to the second molar. Therefore, resulting in excessive stress concentration and undesirable molar distal and buccal tipping and extrusion.
3. Moreover, the stress concentration on the distal miniscrew of the iPanda was six times higher than in the mesial miniscrew with an extrusive and intrusive vector, respectively.

## Data Availability

Not applicable

## Abbreviations

FE: Finite element
FEA: Finite element analysis
FEM: Finite element method
